# Supplementary material for: Genomic and Biological Insights of Bacteriophage ΦBc24 Targeting Bacillus cereus
Source: Curr Issues Mol Biol. 2025 Oct 31;47(11):906. doi: 10.3390/cimb47110906 (PMC12651245; doi:10.3390/cimb47110906)
Supplement: Supplementary file 1 [file cimb-47-00906-s001.zip › cimb-3911579-Supplementary Table S1.pdf]

Supplementary Table 1: Host range of phage ΦBc24.
Different bacterial species were used as the host to determine the host spectrum of the phage using a spot test.

Bacterial species	Strain	Source	Reference	Plaque formation
Bacillus cereus	VTCC 11273	National Center for Microbial Gene Resources, Institute of Microbiology and Biotechnology	N/A	++
	VTCC 10949			+
	VTCC 11289			+
	VTCC 11265			+
Bacillus thuringiensis	HL 1.1	Institute of Biology, VAST	N/A	-
	HL 2.2			-
	HL 1.3			-
Bacillus subtilis	BS_VN01	Honeybees	[34]	-
Bacillus pumilus	BP_AM1	Honeybees	N/A	-
Salmonella enterica	*Salmonella*_HaiDuong1	Diseased chicken	[35]	-
Vibrio parahaemolyticus	Vp-HP1	Diseased shrimp	N/A	-
Escherichia coli	*E. coli*_5	Diseased chicken	[36]	-
Lactobacillus kunkeei	LK_VN01	Honey	[38]	-
Staphylococcus aureus	SA 02	Beef	[37]	-

++: clear plaques, +: faint plaques, -: no plaques formed; N/A: Not available
